# The Cantril Ladder elicits thoughts about power and wealth

**DOI:** 10.1038/s41598-024-52939-y

**Published:** 2024-02-01

**Authors:** August Håkan Nilsson, Johannes C. Eichstaedt, Tim Lomas, Andrew Schwartz, Oscar Kjell

**Affiliations:** 1 Department of Psychology, Lund University, Lund, Sweden; 2 Oslo Business School, Oslo Metropolitan University, Oslo, Norway; 3 Department of Psychology, Institute for Human-Centered A.I., Stanford University, Stanford, CA USA; 4 Department of Epidemiology, Harvard University, Cambridge, USA; 5 Department of Computer Science, Stony Brook University, Stony Brook, NY USA

**Keywords:** Psychology, Human behaviour

## Abstract

The *Cantril Ladder* is among the most widely administered subjective well-being measures; every year, it is collected in 140+ countries in the *Gallup World Poll* and reported in the *World Happiness Report*. The measure asks respondents to evaluate their lives on a ladder from *worst* (bottom) to *best* (top). Prior work found Cantril Ladder scores sensitive to social comparison and to reflect one’s relative position in the income distribution. To understand this, we explored how respondents interpret the Cantril Ladder. We analyzed word responses from 1581 UK adults and tested the impact of the (a) ladder imagery, (b) scale anchors of *worst* to *best* possible life, and c) *bottom* to *top*. Using three language analysis techniques (dictionary, topic, and word embeddings), we found that the Cantril Ladder framing emphasizes power and wealth over broader well-being and relationship concepts in comparison to the other study conditions. Further, altering the framings increased preferred scale levels from 8.4 to 8.9 (Cohen’s *d* = 0.36). Introducing *harmony* as an anchor yielded the strongest divergence from the Cantril Ladder, reducing mentions of power and wealth topics the most (Cohen’s *d* = −0.76). Our findings refine the understanding of historical Cantril Ladder data and may help guide the future evolution of well-being metrics and guidelines.

## Introduction

The Cantril Ladder^1^ is one of the most widely used measures of life satisfaction (i.e., well-being), in which individuals evaluate their lives by imagining a ladder representing their *worst* (bottom) to *best* (top) possible life. The instrument is part of the ongoing Gallup World Poll^2,3^, which has collected more than 2.5 million responses to this question in nearly all countries in the world since 2005 and forms the basis for the annual country well-being rankings in the World Happiness Report^4^. Similarly, increasing numbers of countries are measuring well-being directly^5^, including the UK, Germany, and the US, for which the Cantril Ladder is recommended by non-governmental policy institutions such as the OECD^6,7^. Crucially, however, these developments have unfolded with limited research about (1) how respondents interpret and answer the Cantril Ladder (assuming that they think of well-being) and (2) which level they prefer (assuming everyone prefers the top of the ladder).

The Cantril Ladder is typically used to assess the evaluative component of subjective well-being^4,8–10^, a nominally value-neutral approach in which individuals decide for themselves what well-being is^9^. However, respondents’ ratings on the Cantril Ladder do not convey what they consider in the life evaluation (are they considering joyful experiences, relationships, income, or something else?). Thus, understanding how respondents interpret the Cantril Ladder and their preferred level is essential for understanding what the instrument actually measures. If individuals consistently do not prefer the top of the Cantril Ladder (i.e., the best possible life), as typically assumed in analyses of the scale, it may indicate: (i) a misalignment between the scale's theoretical construct of “the best possible life” and how the scale is interpreted; and/or (ii) a divergence from the conventional assumption that the top of the ladder universally represents an aspiration by all individuals.

At the individual level, the Cantril Ladder correlates more strongly with income than with affective well-being^4,11–13^. The ladder symbol, featured prominently in the measure, has been used to measure subjective social status and one’s position in the societal hierarchy^15^. Aggregated from individual samples to the country level, the Cantril Ladder has also been strongly related to GDP and grouped with a socioeconomic progress factor rather than a *well-being factor* [that includes, for example, smile/laughter, happiness, and purpose in life^14^]. Economic social comparison effects at the individual level, such as being surrounded by richer neighbors, relate to lower well-being^16^. These comparison effects in the income hierarchy at the individual level have been purported to explain why countries have not increased their life satisfaction as their GDPs have grown [the Easterlin Paradox^17,18^]. Understanding the mental process by which individual respondents generate a response on the Cantril Ladder might help explain its strong relationship to income and income comparisons.

Of note, the Cantril Ladder anchor focuses on the "*best possible life"* and does not mention life satisfaction, well-being, or happiness, the concepts it is typically assumed to measure^2,4,6^. The instrument further describes a scale from *bottom* to *top* together with this focus on the *worst* to *best* possible life, which might prompt associations with hierarchy *and power*^19^. Our main objective was to understand how respondents interpret the Cantril Ladder by examining the aspects of the instrument and individuals’ preferred level. In addition, we examined alternative conceptions of subjective well-being, specifically *happiness* and *harmony* [the latter being more highly valued in interdependent cultures^20^]. *Happiness* is the lay term for well-being and typically involves more affect and less life evaluation than life satisfaction^11^. *Harmony in life* reflects inner peace and balance^21–24^ and has been shown to be a distinctive life evaluation approach from life satisfaction^23^, with a larger focus on relationships^23^ and cooperative behavior^25^. It has been among the most common lay definitions of happiness across the world^21,22^, and Gallup started including harmony measures in their world poll in 2020^26,27^.

**Figure 1:**
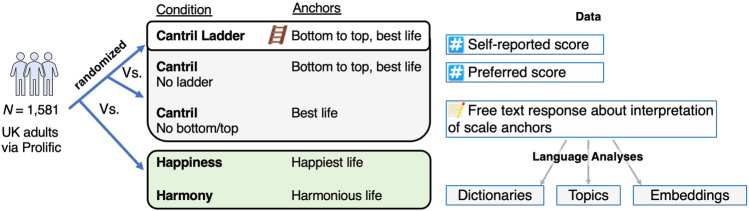
Summary of the study design.

To examine how individuals interpret the Cantril Ladder and their preferred level, we designed two studies in which we experimentally manipulated the three key aspects of the Cantril Ladder, one at a time, that we hypothesized would promote a focus on power and wealth (see Fig. 1 and Table 1). These aspects were examined across five independent randomized groups at the individual level: in the first three groups, we examined the Cantril Ladder by removing aspects of its framing, and in two additional conditions, we examined alternative scale anchor concepts of Happiness and Harmony. Thus, we had the following five conditions: (1) the *Cantril Ladder* (original instrument), (2) the *Cantril no ladder* (the ladder framing removed), (3) the *Cantril no bottom/top* (the ladder and bottom-to-top description removed), as well as scale anchors of (4) *Happiness,* and (5) *Harmony* (rather than best possible life, with the ladder and bottom-to-top description removed). We evaluated the difference in how respondents interpreted the scales by comparing descriptive language responses and preferred levels, which we collected immediately after they had responded to the scales themselves as they are typically used.

The language responses were analyzed using three complementary language analyses covering closed- and open-vocabulary methods to gain a nuanced understanding of language differences and increase the robustness of the findings. The first approach involves counting the relative frequencies of the psychological Power and *Money* dictionaries derived from the *Linguistic Inquiry and Word Count* software [LIWC-2022^28^], which is widely used in psychology. The second approach involves the data-driven modeling of *topics*. Topics cluster semantically related words based on co-occurrences using the probabilistic algorithm Latent Dirichlet Allocation [LDA^29^]. The third approach uses transformer-based^30^ Large Language Models to convert the words in participants’ scale interpretations into *word embeddings—*numeric vectors that encode the words’ meaning^31^. Importantly, embeddings allow us to compute differences in meaning as distances between the language of two conditions. We also use word embeddings to classify the different conditions using logistic ridge regression models and provide examples of language interpretations with the highest probability for each condition.

## Results

**Figure 2:**
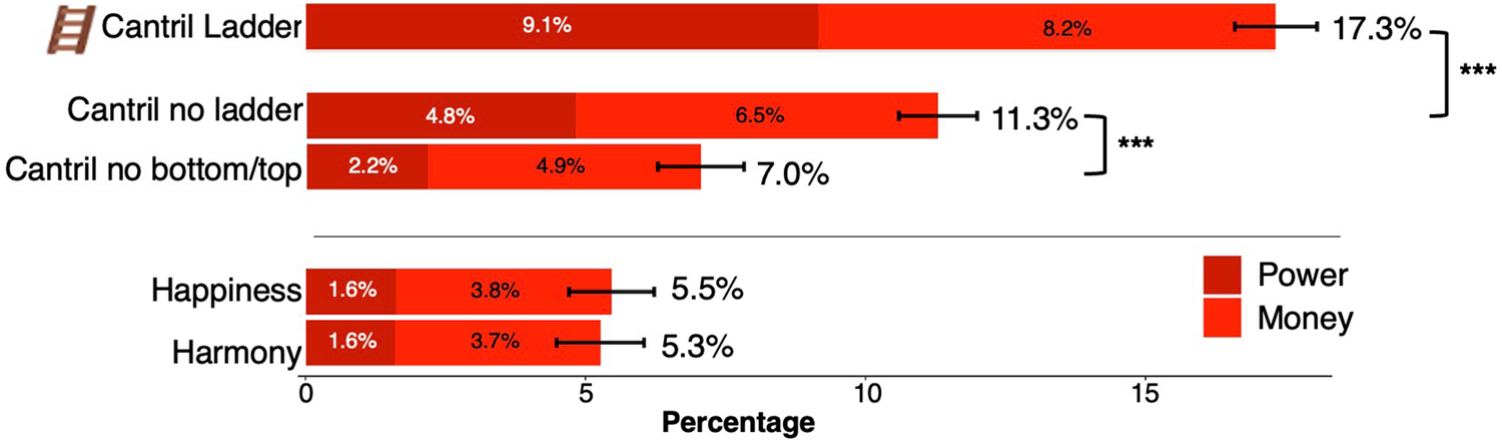
Relative frequency of closed dictionaries for “Power” and “Money”. This figure shows relative frequencies of the Linguistic Inquiry & Word Counts (LIWC) dictionaries Power and Money for the different study conditions, expressed in % of the words used in the interpretations generated by the participants. ***p < 0.001 in pairwise t-tests. Black bars show standard errors.

The ladder and bottom-to-top scale anchor descriptions influenced respondents to use significantly more words from the LIWC dictionaries *Power and Money* when interpreting the Cantril Ladder (Fig. 2) compared to when these anchors were removed. Of all the words respondents used to describe the top of the Cantril Ladder, 17.3% fell into the Power and Money dictionaries. This language was reduced by more than a third when the ladder was removed in the no-ladder condition (absolute difference of 6.0%, *d* = 0.35, *p* < 0.001), and more than halved when the bottom-to-top scale descriptions were removed too (absolute difference of 10.3%, *d* = 0.64, *p* < 0.001). Further, for the Cantril Ladder, words in the Power and Money dictionaries occurred 3.3 times as frequently compared to the alternative Harmony anchor condition (absolute difference of 12%, *d* = 0.77, *p* < 0.001). Thus, the Power and Money dictionaries were more frequent in the Cantril Ladder condition compared to all the other conditions. Still, a majority of the words used to describe the top of the Cantril Ladder condition did not involve Power and Money (or were contained in these LIWC dictionaries).

Removing aspects of the Cantril Ladder reduced *Power* language associations more than *Money* associations. (Fig. 2). Power and Money language were equally common in the Cantril Ladder condition (approx. 50/50), while in the Cantril no bottom/top condition, Money language was more than twice as common as Power language (approx. 70/30).

### The impact of the ladder framing

**Figure 3:**
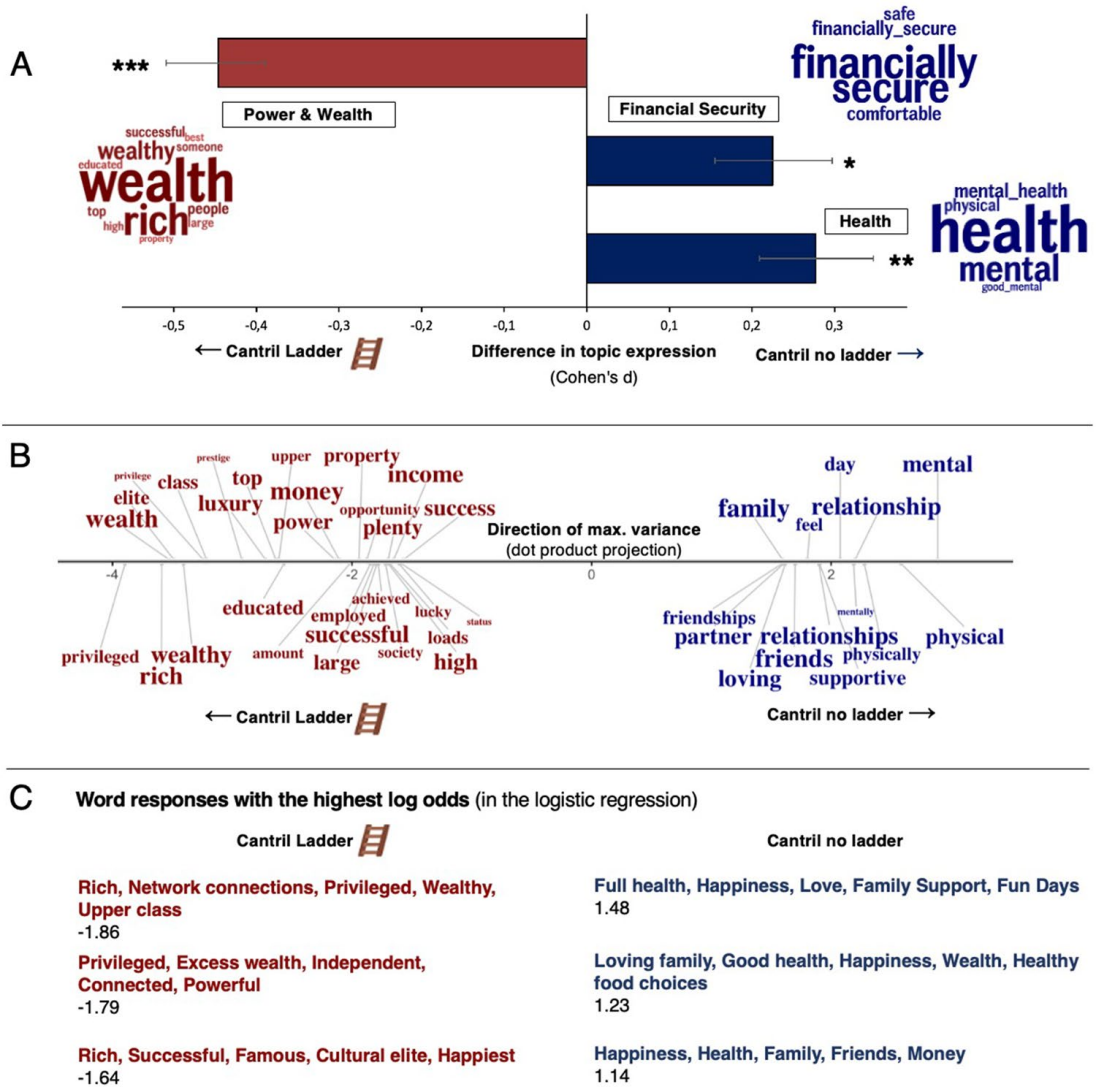
The impact of the ladder: Cantril Ladder (red) vs Cantril no ladder (blue). (**A**) The LDA topics and their mean differences in relative frequencies between the Cantril Ladder versus Cantril no ladder conditions expressed in Cohen’s *d*. (**B**) Significant words related to the Cantril Ladder (red) versus Cantril no ladder (blue) conditions in the word embedding space compared to a permuted null distribution. The font size represents frequency. The position on the x-axis represents the dot product score on the direction line representing the maximal variance. For better visualization, the words are separated on the y-axis, but the y-axis does not represent any information. (**C**) The three scale interpretations with the highest out-of-sample log odds of being in each condition from the logistic ridge regression model.

Open-vocabulary and word embedding-based language analysis further demonstrated that the ladder evoked language related to power and money that focused on wealth, in comparison to the Cantril no ladder condition (Fig. 3). A power and wealth topic, including “best,” “rich,” and “successful”, was significantly more used in the Cantril Ladder condition than in the no-ladder condition (*d* = 0.45, *p* < 0.001). Respondents still focused on money in the no-ladder condition—but they focused more on financial safety, security, and comfort than on wealth and being rich (Fig. 3A). In addition, removing the ladder framing and symbol influenced individuals to focus more on mental and physical health and, from embedding-based analysis, relationships, and family (Fig. 3B). The scale interpretations that the logistic ridge regression model (AUC = 0.62, *p* < 0.001) gave the highest probabilities of being in each condition aligned with the previous results (Fig. 3C). For example, “Rich, Network connections, Privileged, Wealthy, Upper class” had the highest probability of being associated with the Cantril Ladder condition (log odds = −1.86).

### Differences in preferred levels

**Figure 4:**
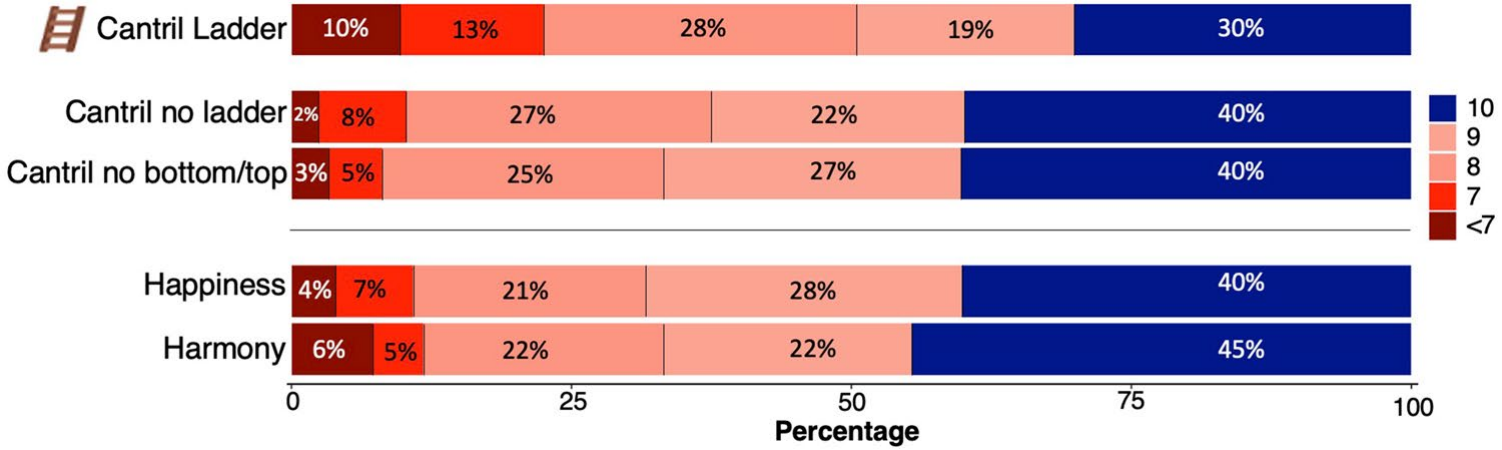
Distribution of preferred levels. The figure shows the distribution of preferred level: all scores below 7 were grouped into one color. The differences between the Cantril Ladder to all the other conditions were statistically significant both by using pairwise Wilcoxon rank-sum tests (all *p* < 0.001 adjusted for multiple comparisons, *r* = 0.16–19) and pairwise t-tests of the estimated marginal means that were adjusted for age, gender, and subjective social status (all *p* < 0.001 adjusted for multiple comparisons, *d* = 0.27–0.39, for more details, see the "Statistical analysis” section).

Those randomly assigned the Cantril Ladder condition preferred a significantly lower level on the 0–10 scale (Fig. 4). Although all conditions asked whether the respondents wanted the best/happiest/most harmonious life, 10 was never the majority response. The average Cantril Ladder preferred level (8.39), however, was significantly lower compared to the condition in which the ladder framing was removed (8.87; *d* = 0.36, *p* < 0.001 using pairwise t-test of the estimated marginal means that were adjusted for age, gender, and subjective social status across all conditions), and significantly lower than all the other conditions using both interval and ordinal tests (*p* < 0.001, see Fig. 4 notes and Table S1 in the Supplementary Information for descriptives). There were no other significant differences in preferred levels between the conditions apart from the Cantril Ladder condition.

### The Cantril Ladder contrasted against harmony scale anchors

**Figure 5:**
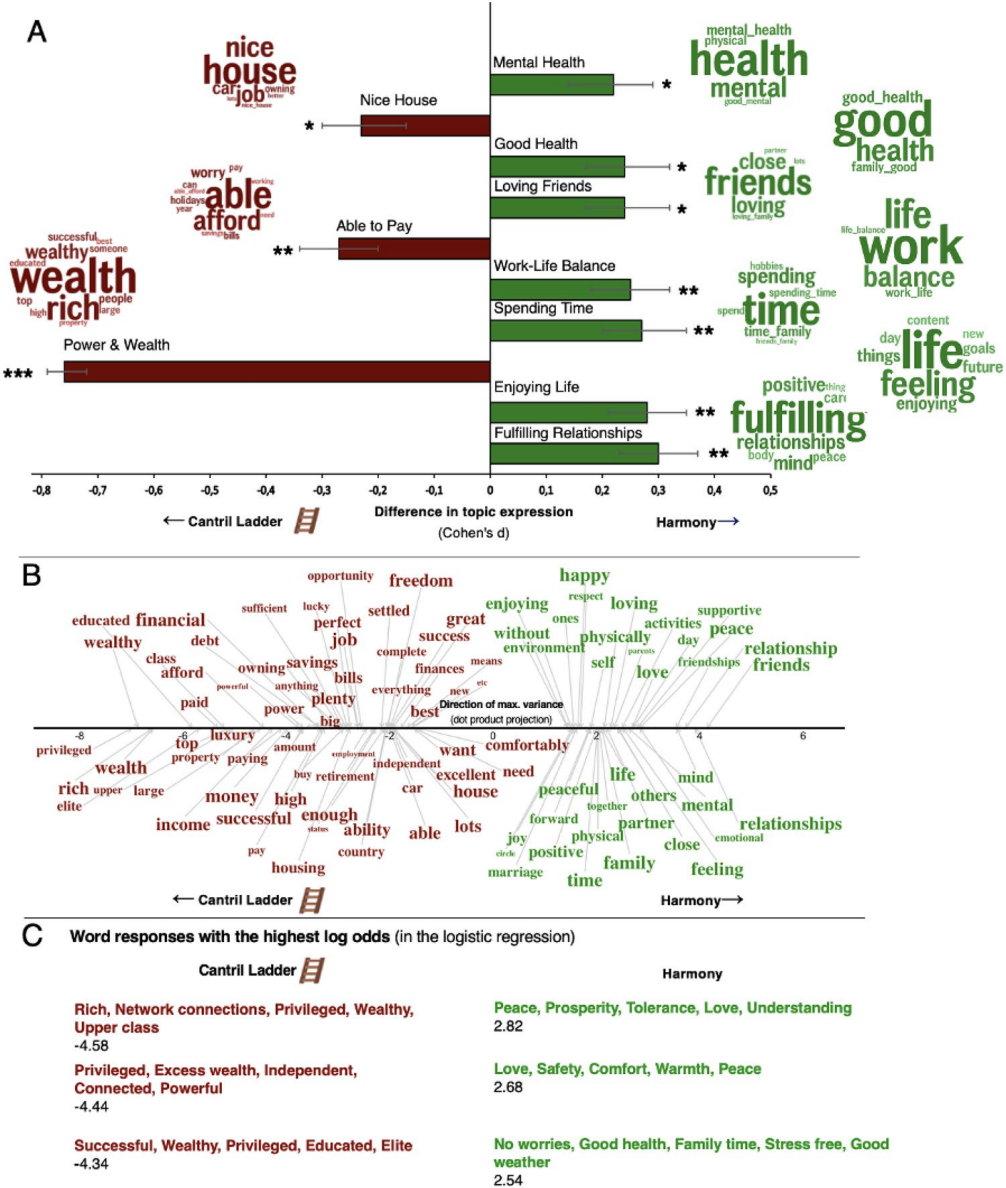
The top of the ladder (red) vs the most harmonious life (green). (**A**) The LDA topics and their mean differences in relative frequencies between the Cantril Ladder and Harmony conditions expressed in Cohen’s *d*. (**B**) Significant words related to the Cantril Ladder (red words) and Harmony (green) in the word embedding space compared to a permuted null distribution. The font size represents frequency. The position on the x-axis represents the dot product score on the direction line representing the maximal variance. For better visualization, the words are separated on the y-axis, but the y-axis does not represent any information. (**C**) The three scale interpretations with the highest log odds of being in each condition from our logistic ridge regression model.

Among conditions, the Harmony scale anchors (“number 10 represents the most harmonious life for you”) showed the most pronounced differences in interpretations compared to the original Cantril Ladder (Fig. 5; for other comparisons between conditions, see Supplementary Information Fig. S1–S7). This comparison foregrounded the predominance of power and wealth associations for the Cantril Ladder, with the corresponding topic (“rich, wealthy”) being substantially more prevalent in the linguistic association (*d* = 0.76, *p* < 0.001; Fig. 5A). On the other hand, relative to the Cantril Ladder, the Harmony anchors elicited linguistic associations focused on relationships (e.g., “fulfilling relationships” , “spending time with family”, “loving friends”) and balanced well-being (“good mental health”, “work-life balance” , “enjoying life”). The scale interpretation associated with the Harmony condition with the highest probability in the logistic ridge regression model (AUC = 0.85, *p* < 0.001) reflected peacefulness and love (log odds = 2.82; “Peace, Prosperity, Tolerance, Love, Understanding”; Fig. 5C).

### Self-reported Cantril Ladder scores related the strongest to subjective social status among the conditions

To further understand the relationship the scales of the study conditions have with power and wealth, we predicted subjective social status, as measured through the MacArthur Ladder^15^, from the self-reported scale scores in each condition, controlled for age and gender (Table S3 in the Supplementary Information). The relationship subjective social status had to the Cantril Ladder scale (β = 0.54, SE = 0.05) was stronger than to the Cantril no ladder (β = 0.43 SE = 0.05), the Cantril no bottom/top scale (β = 0.43 SE = 0.05), the Happiness (β = 0.31, SE = 0.06) and the Harmony (β = 0.35, SE = 0.6) scales. Thus, both the interpretation and the self-reported score of the Cantril Ladder related more strongly to subjective power than the other conditions.

## Discussion

This paper studies how the Cantril Ladder is interpreted by examining the impact of its aspects (invoking a Ladder, scale anchors of “top to bottom” and “best possible life”) on participants' interpretation. First, the results of three different language analysis methods showed that the original Cantril Ladder evokes more connotations of power and wealth *relative to conditions excluding the ladder and re-formulating the scale anchors* and that participants prefer a lower level on the Cantril 0–10 scale than in any other condition. Second, we found that these effects are in large part attributable to the *ladder framing* and the *bottom-to-top* scale anchor descriptions. Third, we discovered that alternative conceptions of well-being—happiness and, particularly, harmony—more strongly than the Cantril Ladder evoked associations of broader notions of well-being, for which participants also preferred higher levels than the Cantril Ladder.

Concerning the stronger connotations of power and wealth in relation to the Cantril Ladder compared to the other conditions, numerous studies have shown the strong relationship the Cantril Ladder has to GDP, income, and other income and wealth-related measures^11–14^. In fact, the cross-country Cantril Ladder distribution has been described as looking “much the same as an income map of the world” (^32^, *p*. 56). In our study, at the between-person level, the Cantril Ladder scale converged more strongly with subjective social status than the scales of the other study conditions (*r* = 0.53). There is consistent research on the importance of income for well-being, but the strength and nature of the relationship (e.g., linear or curvilinear) differ among well-being dimensions and baselines^33,34^. Life satisfaction has shown a stronger relationship with income than emotional well-being, for which the relationship with income is relatively small^4,33–36^. This dovetails with our results: the original Cantril Ladder influenced respondents to focus more on money in terms of wealth (whereas when the ladder framing was excluded, they focused more on financial security) than the other conditions. These results can guide the selection of instruments and the interpretation of the results regarding what function of money is implicitly tapped in the interpretative mind of the respondent (i.e., money for the purpose of wealth vs. financial security). Importantly, measures that capture power and wealth to a large degree can be very useful; for example, socioeconomic status is a strong predictor for health outcomes such as immune system functioning^37^.

Less than half of the participants preferred a score of 9 or 10 on the Cantril Ladder, which is notable. The putatively value-neutral approach of the Cantril Ladder includes instructions that “the top of the ladder represents the best possible life for you,” intending to allow individuals to decide for themselves what the best possible life entails for them (e.g., an optimal balance of happiness, relationships, and challenges). Yet, over 50% did not prefer the highest level (of 10) in any of the study conditions, and less than a third preferred the top of the Cantril Ladder, which had a significantly lower average preferred level than all the other study conditions. It is further notable that respondents preferred a higher level of happiness and harmony, considering that these concepts, in principle, constrain the respondents more than the best possible life (which could include happiness, harmony, and anything they prefer).

The finding that the ladder framing and the bottom-to-top description evoked these effects invites reflection on how life satisfaction and well-being measurement tools are framed. When the ladder framing and the bottom-to-top description were removed, the focus on power and wealth was significantly reduced and replaced by an increased focus on relationships and health, and the preferred level on the scale increased. Of note, the evoked language differences between the Cantril Ladder with and without the ladder were significant but not strong, as measured by a language-based predictive model with an AUC = 0.62. The language differences were more pronounced between the ladder and the harmony condition (AUC = 0.85), reflecting the decreased focus on power and wealth and increased focus on broad well-being (Fig. 5). It is likely that the ladder framing imposes a hierarchical perspective that influences individuals to interpret it as less compatible with other essential aspects of well-being, such as belongingness and mutuality in relationships^21,22,38–42^. These effects could explain why only roughly a third of respondents in the Cantril Ladder condition preferred a “10” despite 10 representing their “best possible life.” Broad well-being (beyond mere status) is a highly desired life goal for society and most individuals^43–45^, which appears to be better captured by the Happiness and Harmony scale anchors.

The most common alternative to the Cantril Ladder for large-scale measurement of life satisfaction at the national level is the single-item life satisfaction question^6^. Since this measure does not invoke a specific symbol or metaphor, it likely avoids some power and wealth connotations associated with the original Cantril Ladder. However, the two measures are generally strongly related [*r* = 0.75^11^], and language analysis of life satisfaction has also shown a strong emphasis on wealth when compared with harmony in life^23^. This study aimed to experimentally manipulate one aspect of the Cantril Ladder at a time, but future research could employ a similar method to understand the similarities and differences between the single-item life satisfaction and the Cantril Ladder or other impactful well-being scales.

Given that our experimental studies focused on the UK population, these results might apply to similar Western populations and cultures. Although future studies need to confirm the findings in Western contexts, it is in non-Western cultures where these findings may prove even more potent, as harmony, modesty, and non-materialistic values often play a more central role outside Western cultures^46,47^. Further, the results are based on respondents’ interpretation of the scales—future research could examine to what extent these interpretations dovetail with observable real-world behaviors and outcomes. More generally, the findings presented here stress the importance of carefully “focus grouping,” cognitive testing^48^, or otherwise attending to the interpretation that particular choices of framing and phrasing of survey scales elicit in the minds of respondents, with a particular need to take cross-cultural differences in interpretations into account if the resulting data is meant to be comparable across them^49^. Our results suggest that contemporaneously collecting participants’ interpretations of the meaning of survey scales as short text responses—in conjunction with participants’ preferred levels—may provide a cost-effective means to capture the *cognitive and interpretative field of* survey questions for particular samples.

### Limitations

The Cantril Ladder evoked more connotations of power and wealth only in comparison to the other study conditions. Apart from the other conditions, there is no other proper baseline to compare the 17.3% LIWC Power + Money in the Cantril Ladder against. This is because the response format in this study asked for short descriptions, which makes respondents use a different language compared to other domains where there exist clearer baselines (such as Tweets and natural conversations^26^). Thus, the results only show that the Cantril Ladder evokes more power and money in comparison to the other study conditions.

The results indicate that the Cantril Ladder has a lower preferred level than the other conditions. While the language-based results for the Cantril Ladder condition suggest more focus on power and wealth, none of the results provide a clear explanation as to why less than half the respondents in any condition preferred the best/happiest/most harmonious life. We can likely rule out unserious responses considering we included an attention check, it was a short data collection (no fatigue), and the language responses yielded results with high face validity. Future research is needed to understand why the majority of respondents do not prefer the highest score across conditions.

## Conclusion

We show how the phrasing and framing of the Cantril Ladder influence individuals to consider power and wealth more and relationships less compared to conditions excluding the ladder and reformulating the scale anchors. This was supported by three complementary language-based analysis methods relying on both theory-based dictionaries and open-vocabulary exploration. We further show that the ladder framing influences individuals to prefer a lower level on the Cantril Ladder’s 0–10 scale compared to conditions where the hierarchical anchors are removed or replaced with broader conceptions of well-being (happiness or harmony). The Cantril Ladder is arguably the most prominent measure of well-being, but the results suggest caution in its interpretation—the Cantril Ladder’s structure appears to influence participants to attend to a more power- and wealth-oriented view of well-being.

## Methods

### Data collection

The data were collected from two studies featuring a similar design, with the different study conditions as the only difference. The first study’s data collection occurred on February 1st, 2022, between 6 and 8 pm local time and involved the *Cantril Ladder* and *Cantril no ladder* conditions. The second study’s data was collected on March 1st, 2022, between 1 and 3 p.m. local time. Participants were not allowed to partake in both studies. Otherwise, the studies were similar, only differing in three additional questions reported *after* the study variables presented in this paper. Individuals were asked on Prolific (prolific.co), an online platform enabling the recruitment of participants that also provides demographic data [for Prolific’s use in academia, see^50^], to partake in a study regarding their lives and well-being. After being directed to the survey, they were informed about the study and had to provide their consent to participate (see Ethical statement below). The participants were randomly assigned to one of the Cantril variants for which (i) they self-reported their well-being, (ii) provided five brief text descriptions of their impressions of the upper and lower ends of the scale, and (iii) their preferred levels. Participants used 3.15 words (SD = 2.58) per description (more descriptives about the words can be found in Supplementary Information, Table S2).

### Participants

1595 UK adults were recruited from Prolific. Of these, 12 failed to correctly answer an attention check (see Supplementary Information) and were, together with two who only answered the open-ended questions with numbers, excluded from further analyses, remaining with 1581 participants. In the first data collection, 329 were randomly assigned to the *Cantril Ladder* condition and 361 to the *Cantril no ladder* condition. In the second data collection, 298 were randomly assigned to the *Cantril no bottom/top* condition, 304 to the *happiness* condition, and 289 to the *harmony condition*. We applied screening information that participants had provided at Prolific registration to assess the sample characteristics. 50.2% of participants were male, with a mean age of 39 (*SD* = 14) years and a range of 18–82. For employment status, 49% worked full-time, 21% worked part-time, 7% were unemployed, and 23% had another employment status, such as non-paid work. Apart from employment status, 15% were students. The average subjective social status was 5.28 (*SD* = 1.58), where participants placed themselves on a ladder representing how people stand in society from 1 to 10 [*worst off* to *best off* (i.e. the MacArthur scale of subjective social status^15^). Participants in the first, slightly longer, data collection were compensated £0.50 for the study, which required a median of 5.22 (*SD* = 3.52) minutes to complete, and participants in the second data collection were compensated £0.35 for the study, which required a median of 3.28 (*SD* = 2.55) minutes to complete. The groups did not significantly differ in age and subjective social status in one-way ANOVAs nor with respect to sex, employment status, or student status in chi-square tests of independence (*p* > 0.05).

### Survey instruments

The different Cantril Ladder variants, i.e., the study conditions, are outlined in Table 1. All scale interpretation questions started with ''Considering the question on the previous page (also shown above):'' and had the condition’s scale question on top of the page. The experimental approach aimed to manipulate one aspect at a time (e.g., from “best possible life” to “happiest possible life”). On the open-ended questions, participants were instructed to “Please answer by writing five short descriptions in the response boxes”. Describing various mental health conditions, including well-being, has worked as well with five as with ten descriptions^51^ or a paragraph^52^ in predictive accuracy, and five short descriptions have worked well to provide visual language insights on everyday activities’ relationship to well-being^53^.

**Table 1:**
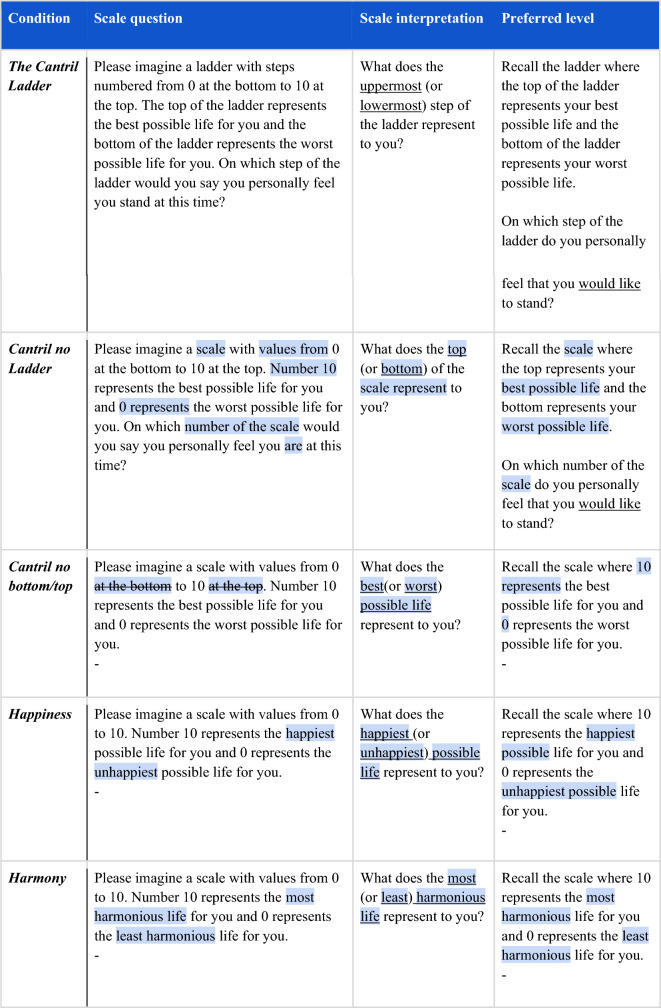
The Cantril Ladder versions.

The text crossed out in blue differs from the previous question. The scale and preferred level questions in the last three conditions were identical. This is indicated by “-”.

### Ethical statement

The studies were conducted from Sweden and complied with Swedish laws and research ethical guidelines and regulations. The Swedish National Ethical Board exempts ethical approval from a study not associated with psychological or physical harm risks, not intended to manipulate/influence participants, nor include any collection of sensitive personal information. Hence, the Swedish National Ethics Review Board has deemed this type of survey and method exempt from needing ethical approval according to Swedish law (see 3–4 §§ law 2003:460 on ethical review of research involving humans; in ethical application 2020-00730 this type of study focusing on depression and anxiety was exempt by the Swedish National Ethics Board). In the consent form, participants were first told about the study, given the researchers' contact information, told about their right to withdraw at any time without giving any reasons, and told that no personally sensitive information would be collected. They were then asked to provide their informed consent to participate and debriefed about the study when they completed it.

### Language-based analysis

#### Linguistic inquiry and word count dictionaries

Linguistic Inquiry and Word Counts (LIWC) include dictionaries with psychological meanings. These dictionaries have been extensively validated since the first version in 2001^28,54^ and are widely used in psychological language analysis. We analyzed the relative frequency in each condition of the Power dictionary, including words such as “own,” “order,” and “allow,” and the Money dictionary, including words such as “business,” “pay,” “price,” and “market”.

#### Latent Dirichlet allocation topic dictionaries

We used the Latent Dirichlet Allocation (LDA) algorithm^29^ to create all the topic dictionaries. Topics are semantically coherent clusters of n-grams (i.e., *n* consecutive words) that co-occur together, where each topic includes weights for the most important n-grams for that topic. We used all the participant’s free text response interpretations of the upper part of all five conditions’ scales to create a total of 30 topics while excluding stop words defined by the Snowball lexicon^55^ as well as the most frequent word because LDA does not model the head of the Zipfian distribution well^56,57^. We used 1-g and 2-g to create the 30 topics and iterated using Gibb's Sampling to fit the LDA model using 20,000 iterations. We chose 30 topics following the rule of thumb to have around 50 documents/topics given the ~ 1500 free text responses in the total sample^56^. The authors created the presented topic names.

#### Word embeddings

All word-related results (i.e., Figs. 3B, C, 5B, C) used Large Language Model-based word embeddings. Word embeddings are numerical representations of words that have been shown to capture the latent meaning of words^31,58^. Large Language Models are models pre-trained on a large amount of text data from the internet (which are the type of models behind all modern chatbots such as BARD and ChatGPT) and are used to convert the text data to word embeddings. For plotting words on the direction of maximal variance, we used the language model BERT *base*^58^, layer 11 (for more details, see^59^). For the classification tasks, we used RoBERTa Large, layer 23^60^, a model that has been especially efficient for predictive tasks^61^ but has extremely skewed distributions that are inappropriate for geometric plots^62^. We excluded stopwords using the Snowball dictionary^55^ for the word embeddings used to plot words on the direction of maximal variance, as stopwords have minimal information content^63^. We aggregated multiple-word embeddings using their mean to represent a respondent's entire scale interpretation.

#### Statistical information

For all statistical analyses, the alpha level was set to 0.05.

#### Hypothesis-driven (LIWC)

For the LIWC analysis visualized in Fig. 2, we used the sum of the raw relative frequencies of the Power and Money dictionaries as the unit of analysis, without any pre-processings, simply using the count of word mentions from the dictionary divided by total word mentions. To control for subjective social status, age, and gender, we added them as covariates with the Power and Money sum as the criterion variable in a linear regression model. A second model added (all) study conditions. While the demographic model was significantly more accurate than just the mean (*F* = 6.42 (3, 1576), *p* < 0.001, R squared = 0.01), the latter model with the conditions included was significantly more accurate than the former (*F* = 47.0 (4, 1572), *p* < 0.001, R squared = 0.11). From this model, we computed estimated marginal means and standard errors in each condition that adjusted any difference between the groups in subjective social status, gender and age. With these adjusted means, we significance-tested the differences by pairwise t-tests while controlling for multiple comparisons of the different conditions using Benjamini-Hochberg’s^64^ correction—these percentages and significance test results are the ones depicted in Fig. 2. We used Cohen’s *d* of the unadjusted means for effect size since it is relatively easily interpretable. Cohen's *d* is a standardized measure that quantifies the difference between two groups' means in terms of standard deviations. Cohen’s *d* is not a multi-variate metric that can be used together with control variables.

#### Data-driven (LDA)

We also used pairwise comparisons and Cohen’s *d* for differentiating language by topics, visualized in Figs. 3A and 5A. To control for subjective social status, gender, and age, we ran multi-variance logistic ridge regression and computed *p*-values from this. Subsequently, we ran the topics in two regression models with the two comparison conditions as the outcome. The models with the demographic variables were never significant (p > 0.05), while the topics were significant predictors in both Fig. 3A (p < 0.001, R squared = 0.04) and Fig. 5A (p < 0.001, R squared = 0.18). The reported topics were significant predictors while correcting for multiple comparisons of the 30 topics using Benjamini-Hochberg’s^64^ correction.

#### Using word embeddings to quantify semantic differences

We used word embeddings to depict the words significantly belonging to one of two groups in the embedding space^51^, depicted in Figs. 3B and 5B. To create the word plots, we compared each word to the distance between the two groups’ aggregated word embeddings (e.g., when comparing the interpretations in the Cantril Ladder condition with those in the harmony condition). Here, we first aggregated all the word embeddings from each group into two aggregated embeddings (one per condition). The first aggregated embedding is subtracted from the other, becoming an *aggregated direction embedding.* This forms a direction line and represents the average word embedding of the words on a linear scale and encapsulates the maximum variance of the language in the two conditions. We projected individual words’ word embeddings to the line using the dot product. To conduct a significance test, we permuted a null distribution (*n* = 50,000) of dot product projections by randomly swapping words from the two conditions when creating permuted aggregated direction embeddings and randomly drawing word embeddings from both conditions. These dot product projections were compared to the null distribution of permuted dot products to yield *p*-values while correcting for multiple comparisons using Benjamini–Hochberg’s correction [^64^ for details, see *Supervised Dimension Projection Plots* in^51^]. Words had to appear at least five times among all descriptions that are compared to be included for each condition comparison.

#### Predictive modeling: classification

We used machine learning and trained binary logistic ridge regression models to classify two conditions based on the word embeddings from each condition. We applied tenfold nested cross-validation to avoid overfitting [as in^51^]: The data was first split into ten random folds. Then, logistic ridge regression models with ridge penalty (ranging 10^seq(−16, 16) and their hyperparameter were computed with the data in nine out of tenfolds and subsequently evaluated in the remaining hold-out fold. We repeated this procedure ten times to estimate out-of-sample probabilities for each participant’s interpretation and converted these probabilities to log odds, shown in Figs. 3C and 5C. Log odds are converted probabilities that typically range between −4.6 and 4.6 (when the probability range is 1 and 99%) but can theoretically reach infinity. We applied AUC to evaluate the final logistic ridge regression model. AUC evaluates all possible combinations of true and false positive rates by varying the threshold of the probabilities. This procedure forms a ROC curve, and the AUC is the *area under this ROC curve*.

#### Preferred levels

For the preferred levels, we also made a control linear regression model with subjective social status, age, and gender as the predictors, and in a second model, we added (all) study conditions, similar to the LIWC analysis. The model, including the conditions, was significantly more accurate than the model with only the controls (*F* = 7.6 (4, 1572), *p* < 0.001, R squared = 0.05). From this model, we computed estimated marginal means in each condition that adjusted the means for any difference between the groups in subjective social status, gender, and age. With these adjusted means, we significance-tested the differences by pairwise t-tests while controlling for multiple comparisons using Benjamini-Hochberg’s^64^ correction. We used pairwise Cohen’s *d* of the unadjusted means for effect size. Since the preferred levels were highly skewed (Table S1 in Supplementary Information), we also conducted an ordinal analysis by a Kruskal Wallis test, which was significant (chi^2^ = 33.3 (4), *p* < 0.001) and used pairwise Wilcoxon rank-sum tests with Benjamini-Hochberg’s^58^ adjustment for multiple comparisons. These pairwise significance tests were identical to those reported in conjunction with Fig. 3, i.e., all conditions had significantly higher medians than the Cantril Ladder, and the effect sizes ranged from *r* = 0.16 to 0.19.

#### Subjective social status and self-reported scores

Also for subjective social status we made a control linear regression model with age and gender as the predictors in the first step, and then adding the self-reported scale scores in the second model. We did this for every condition separately. We standardized all variables before running these regressions and reported the self-reported score’s beta coefficients and standard error. In all conditions, adding self-reported scores significantly improved the models’ accuracies (p < 0.001) based on ANOVA comparing the two models.

### Supplementary Information

Supplementary Information.

## Data Availability

The raw data, processed data, and code are available here. The LIWC analyses were computed with the LIWC 2022 software. The open-vocabulary topic analyses using LDA were done using the Python package DLATK, which uses Mallet. The remaining language analyses were done in R, using RStudio and the text package. Parts of the R computations were enabled by resources provided by the Swedish National Infrastructure for Computing (SNIC) at Chalmers University of Technology. All statistical and computational r packages are mentioned in the Supplementary Information.
