# Supplementary material for: The Cantril Ladder elicits thoughts about power and wealth
Source: Sci Rep. 2024 Feb 1;14:2642. doi: 10.1038/s41598-024-52939-y (PMC10834405; doi:10.1038/s41598-024-52939-y)
Supplement: Supplementary file 1 — Supplementary Information. [file 41598_2024_52939_MOESM1_ESM.docx]

**Supplementary Information for**

The Cantril Ladder Elicits Thoughts about Power and Wealth

August Håkan Nilsson*^1,2^, Johannes C. Eichstaedt^3^, Tim Lomas^4^, Andrew Schwartz^5^, & Oscar Kjell^1^

^1^ = Department of Psychology, Lund University, Sweden

^2^ = Oslo Business School, Oslo Metropolitan University, Oslo, Norway

^3^ = Department of Psychology & Institute for Human-Centered A.I., Stanford University, Stanford, California, USA

^4^ = Department of Epidemiology, Harvard University, USA

^5^ = Department of Computer Science, Stony Brook University, Stony Brook, New York, USA

ment of Computer Science, Stony Brook University, Stony Brook, New York, USA

Corresponding author: August Håkan Nilsson

**Email:** [august.nilsson@psy.lu.se](mailto:august.nilsson@psy.lu.se)

**This PDF file includes:**

Figures S1 to S9

Tables S1 to S3

Supplementary methods

SI References

**Table S1.** *Descriptive statistics for the preferred scores.*

| **Condition** | **Median** | **Mean** | ***SE*** | **SD** | **Skew** | **Kurtosis** | **Range** | **N** |
| --- | --- | --- | --- | --- | --- | --- | --- | --- |
| ***The Cantril Ladder*** | 8 | 8.39 | 0.08 | 1.53 | -1.15 | 1.94 | 1-10 | 329 |
| ***Cantril no ladder*** | 9 | 8.87 | 0.06 | 1.18 | -1.19 | 2.57 | 3-10 | 361 |
| ***Cantril no bottom/top*** | 9 | 8.92 | 0.07 | 1.22 | -2.02 | 9.36 | 0-10 | 298 |
| ***Happiness*** | 9 | 8.90 | 0.09 | 1.26 | -1.66 | 4.49 | 2-10 | 304 |
| ***Harmony*** | 9 | 8.81 | 0.07 | 1.59 | -2.13 | 5.75 | 1-10 | 289 |

**Table S2.** *Descriptive statistics for the text variables/per description.*

| **Condition** | **Mean** | **Median** | **SD** | **Range** | **Unique tokens** | **Entropy** | **N** |
| --- | --- | --- | --- | --- | --- | --- | --- |
| ***The Cantril Ladder*** | 2.92 | 2 | 2.40 | 1 - 18 | 995 | 8.16 | 329 |
| ***Cantril no Ladder*** | 3.17 | 2 | 2.88 | 1 - 33 | 956 | 7.91 | 363 |
| ***Cantril no bottom/top*** | 3.53 | 3 | 2.67 | 1 - 21 | 855 | 7.84 | 298 |
| ***Happiness*** | 3.75 | 3 | 2.90 | 1 - 29 | 920 | 7.93 | 304 |
| ***Harmony*** | 3.39 | 3 | 2.81 | 1 - 29 | 908 | 8.03 | 289 |

**Fig S1. Cantril Ladder (red) vs Cantril no bottom/top (green)**

*
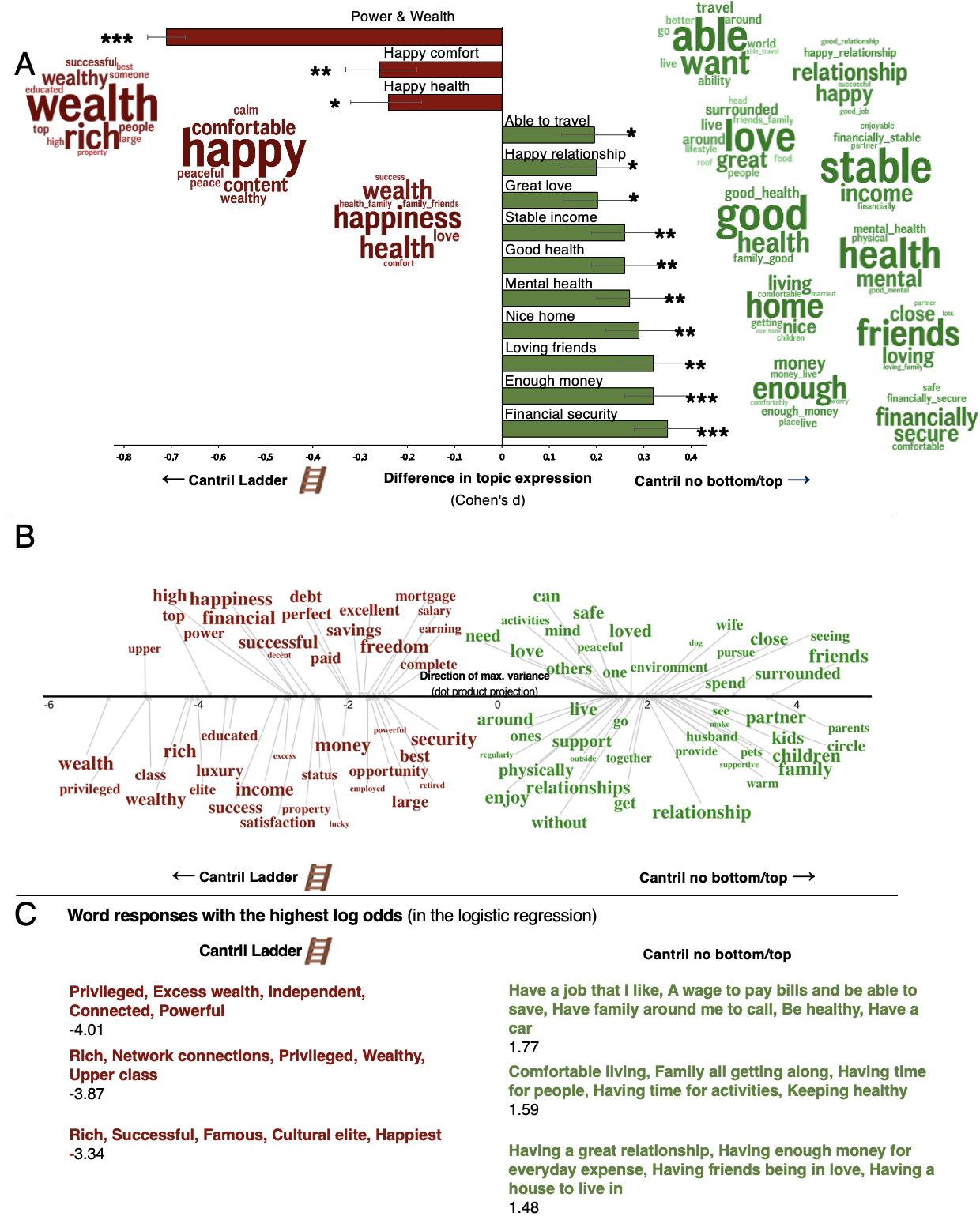
*

*Note:* **Fig. *S1A*** shows the word clusters (LDA topics) and their mean differences in relative frequencies between the Cantril Ladder and Cantril no bottom/top conditions expressed in Cohen’s *d*. **Fig. *S1B*** shows significant words related to the Cantril Ladder (red words) and Cantril no bottom/top (green) conditions in the word embedding space compared to a permuted null distribution. The font size represents frequency. The position on the x-axis represents the dot product score on the direction line representing the maximal variance. For better visualisation, the words are separated on the y-axis, but the y-axis does not represent any information. **Fig. *S1C*** shows the three scale interpretations with the highest log odds of being in each condition from our logistic ridge regression model. The classification model reached an AUC of .74.

**Fig S2. Cantril Ladder (red) vs. Happiness (green)**

**
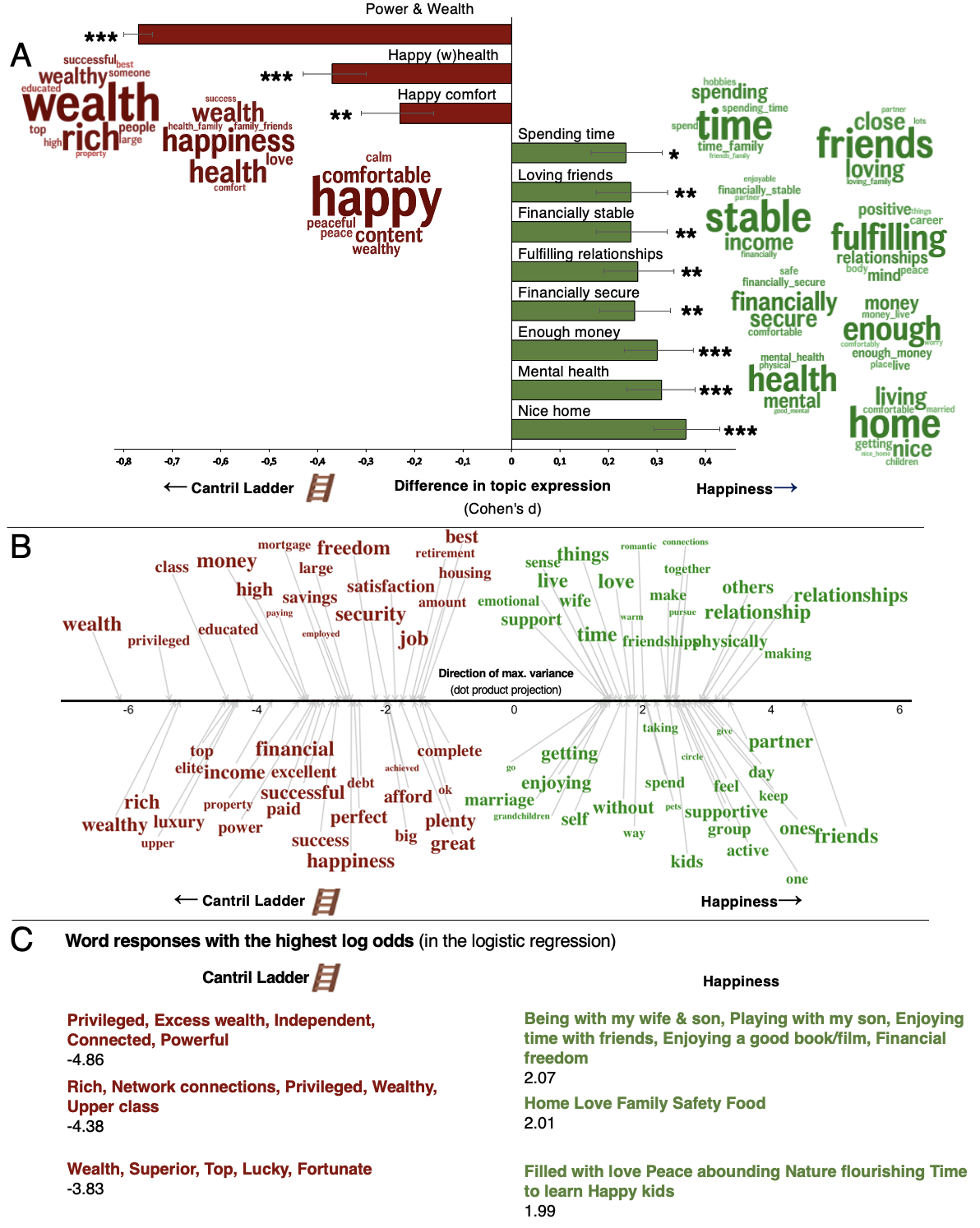
**

*Note:* **Fig. *S2A*** shows the word clusters (LDA topics) and their mean differences in relative frequencies between the Cantril Ladder and Happiness conditions expressed in Cohen’s *d*. **Fig. *S2B*** shows significant words related to the Cantril Ladder (red words) and Happiness (green) conditions in the word embedding space compared to a permuted null distribution. The font size represents frequency. The position on the x-axis represents the dot product score on the direction line representing the maximal variance. For better visualisation, the words are separated on the y-axis, but the y-axis does not represent any information. **Fig. *S2C*** shows the three scale interpretations with the highest log odds of being in each condition from our logistic ridge regression model.. The classification model reached an AUC of .81.

**Fig S3. Cantril no ladder (red) vs. Cantril no bottom/top (green)**

**
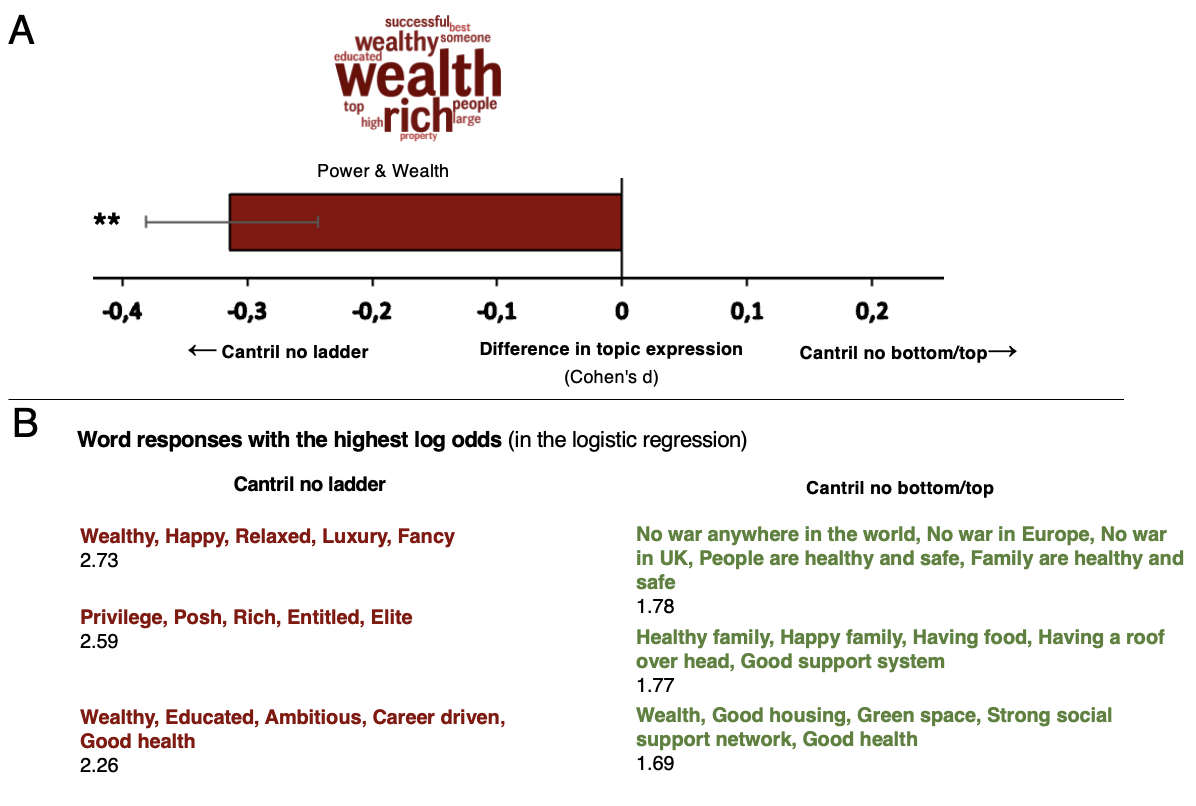
**

*Note:* **Fig. *S3A*** shows the word cluster (LDA topic) and its difference in relative frequency between the Cantril no ladder and Cantril no bottom/top conditions expressed in Cohen’s *d*. **Fig. *S3B*** shows the three scale interpretations with the highest log odds of being in each condition from our logistic ridge regression model. The classification model reached an AUC of .65. No words were significant in the word embedding space after correction for multiple comparisons

**Fige S4. Cantril no bottom/top (red) vs Harmony (green)**

**
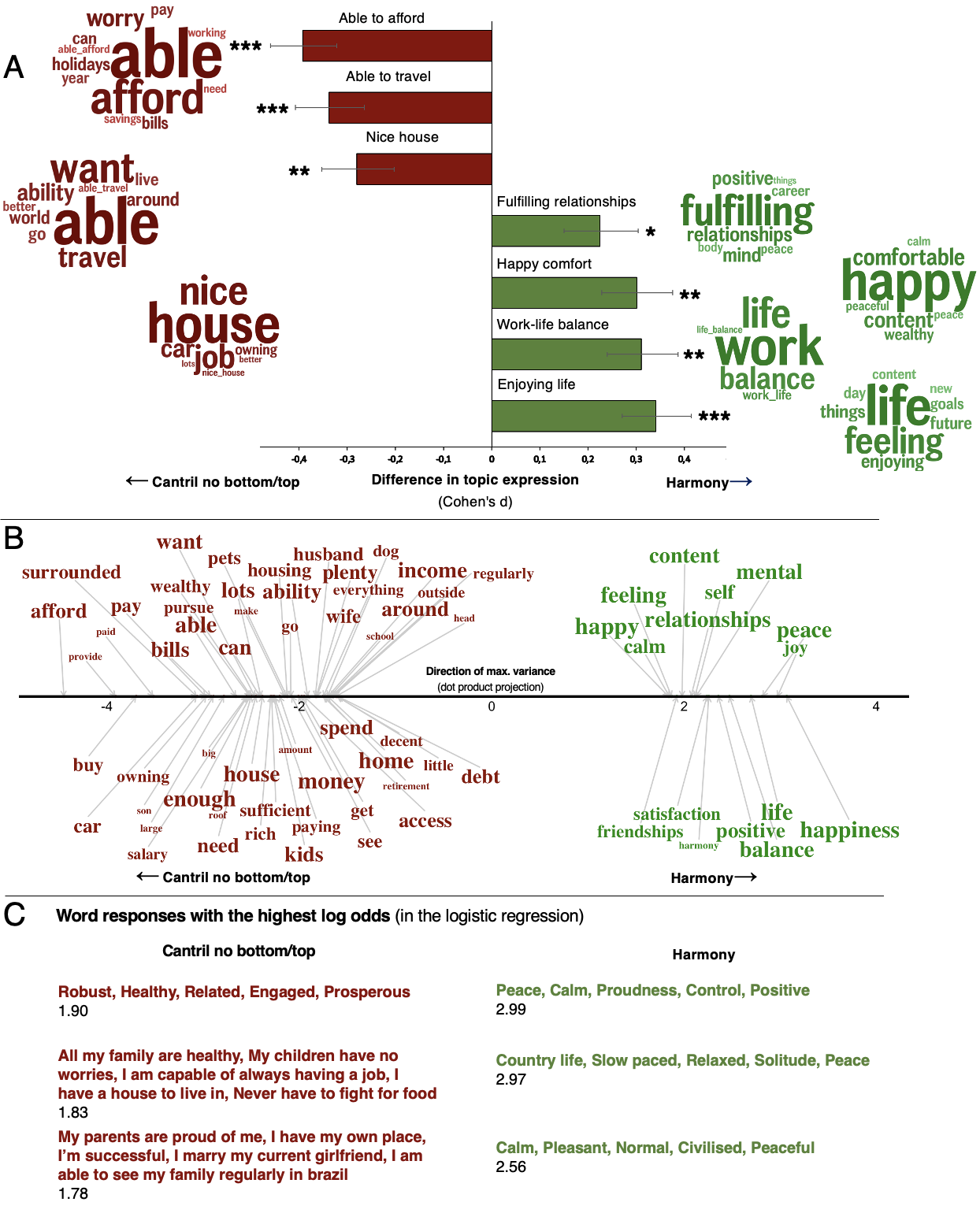
**

*Note:* **Fig. *S4A*** shows the word clusters (LDA topics) and their mean difference in relative frequency between the Cantril no bottom/top and Harmony conditions expressed in Cohen’s *d*. **Fig. *S4B*** shows significant words related to the Cantril no bottom/top (red words) and Harmony (green) conditions in the word embedding space compared to a permuted null distribution. The font size represents frequency. The position on the x-axis represents the dot product score on the direction line representing the maximal variance. For better visualisation, the words are separated on the y-axis, but the y-axis does not represent any information. **Fig. *S4C*** shows the three scale interpretations with the highest log odds of being in each condition from our logistic ridge regression model. The classification model reached an AUC of .70.

**Fige S5. Cantril no ladder (red) vs Happiness (green)**


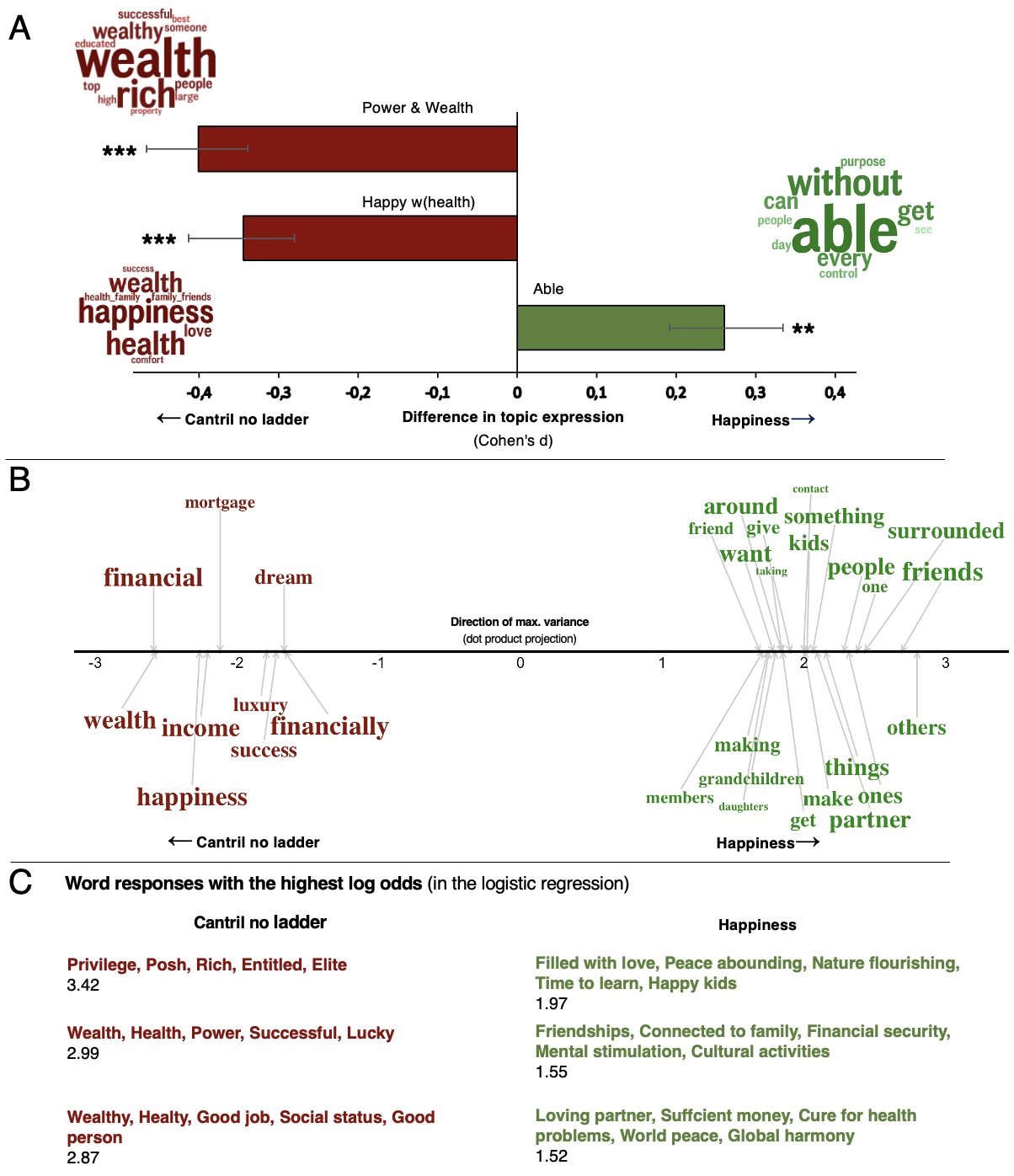


*Note:* **Fig. *S5A*** shows the word clusters (LDA topics) and their mean differences in relative frequency between the Cantril no ladder and Happiness conditions expressed in Cohen’s *d*. **Fig. *S5B*** shows significant words related to the Cantril no ladder (red words) and Happiness (green) conditions in the word embedding space compared to a permuted null distribution. The font size represents frequency. The position on the x-axis represents the dot product score on the direction line representing the maximal variance. For better visualisation, the words are separated on the y-axis, but the y-axis does not represent any information. **Fig. *S5C*** shows the three scale interpretations with the highest log odds of being in each condition from our logistic ridge regression model. The classification model reached an AUC of .69.

**Fig S6. Cantril no ladder (red) vs. Harmony (green)**

**
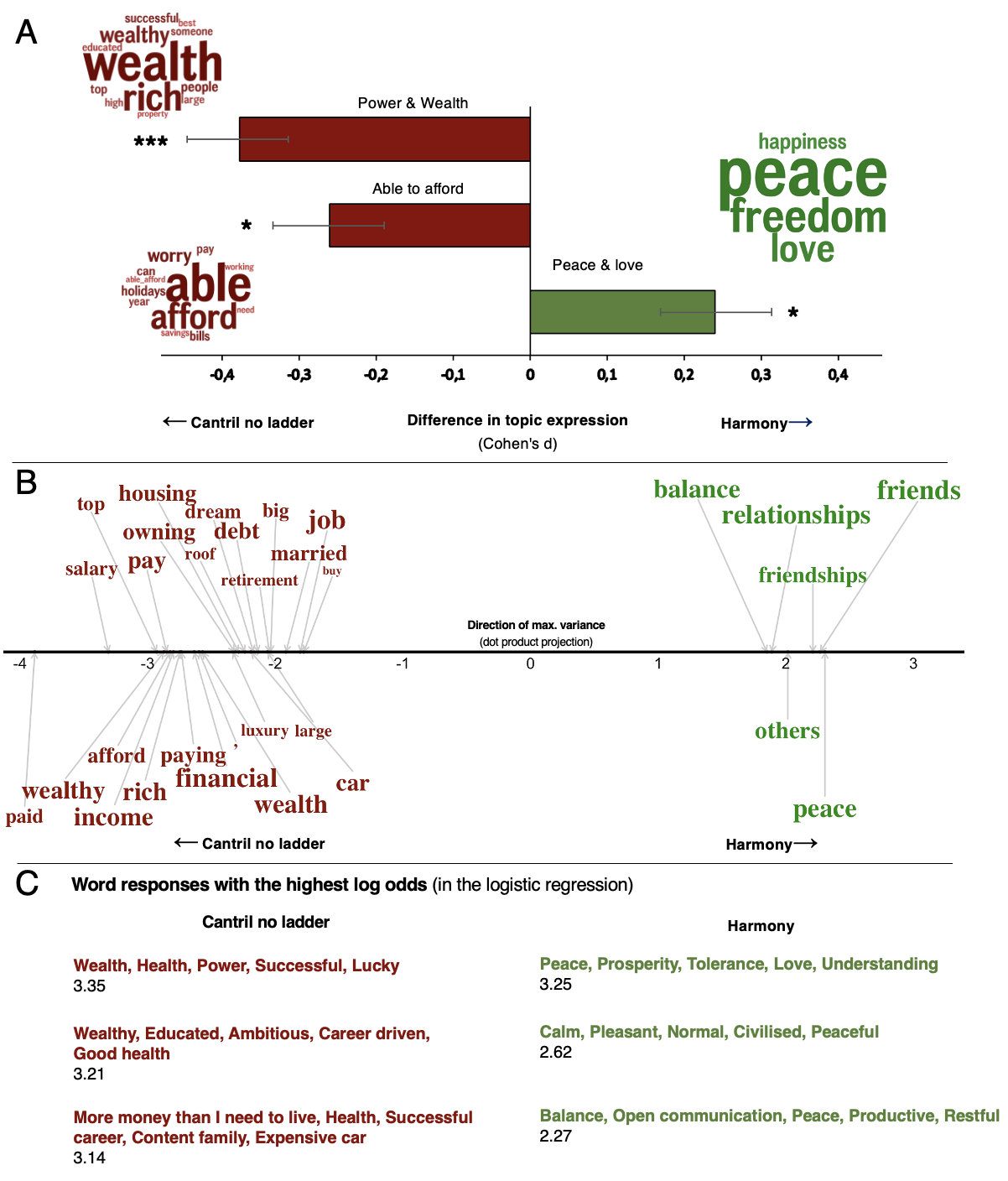
**

*Note:* **Fig. *S6A*** shows the word clusters (LDA topics) and their mean differences in relative frequency between the Cantril no ladder and Harmony conditions expressed in Cohen’s *d*. **Fig. *S6B*** shows significant words related to the Cantril no ladder (red words) and Harmony (green) conditions in the word embedding space compared to a permuted null distribution. The font size represents frequency. The position on the x-axis represents the dot product score on the direction line representing the maximal variance. For better visualisation, the words are separated on the y-axis, but the y-axis does not represent any information. **Fig. *S6C*** shows the three scale interpretations with the highest log odds of being in each condition from our logistic ridge regression model. The classification model reached an AUC of .75.

**Fig S7. Happiness (red) vs Harmony (green)**

**
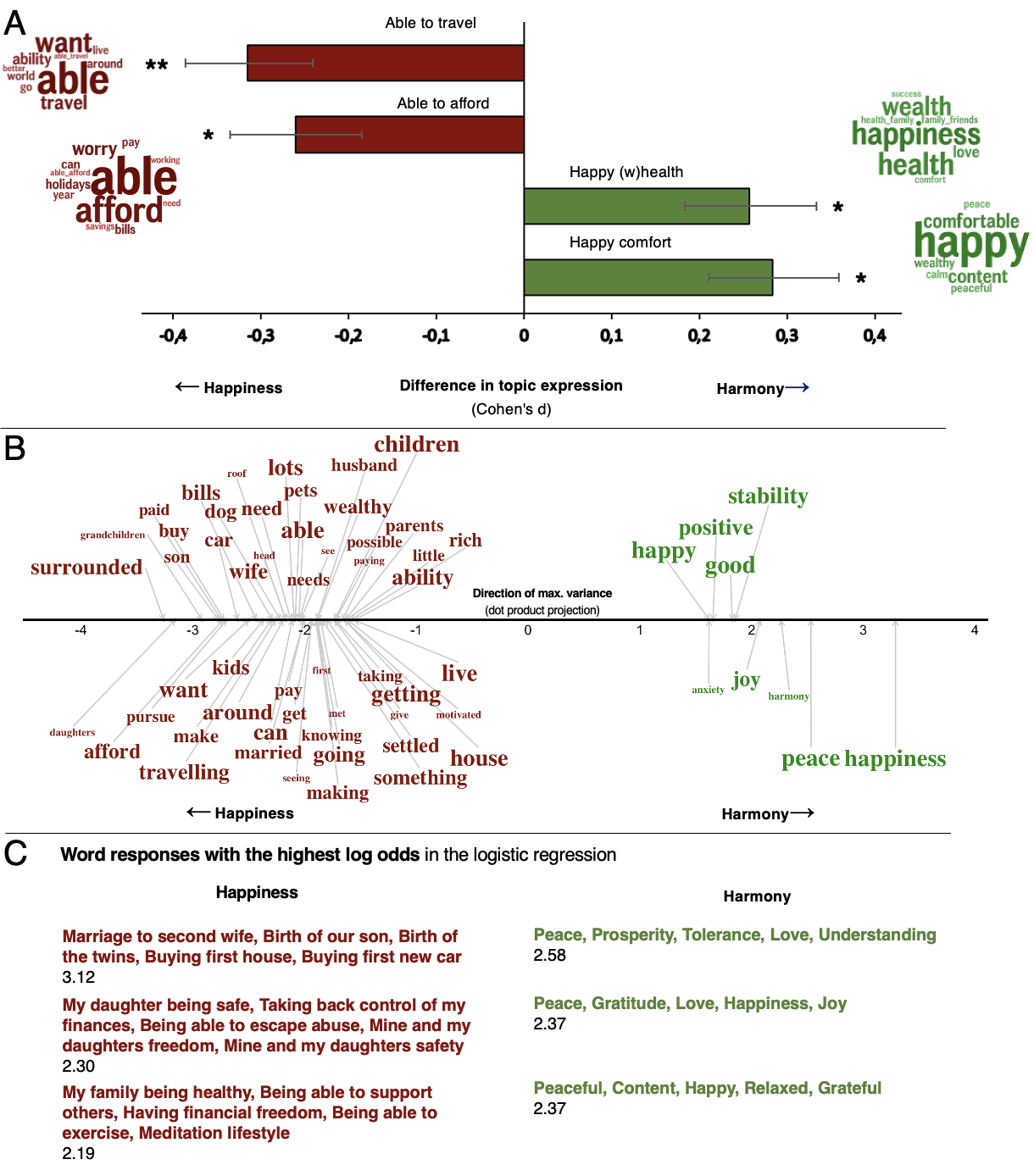
**

*Note:* **Fig. *S7A*** shows the word clusters (LDA topics) and their mean differences in relative frequency between the Happiness and Harmony conditions expressed in Cohen’s *d*. **Fig. *S7B*** shows significant words related to the Happiness (red words) and Harmony (green) conditions in the word embedding space compared to a permuted null distribution. The font size represents frequency. The position on the x-axis represents the dot product score on the direction line representing the maximal variance. For better visualisation, the words are separated on the y-axis, but the y-axis does not represent any information. **Fig. *S7C*** shows the three scale interpretations with the highest log odds of being in each condition from our logistic ridge regression model. The classification model reached an AUC of .67.

**The best possible life with no top formulation vs the happiest possible life had no significant differences.**

**Table S3. Scale score relationships with Socioeconomic status and well-being.**

**
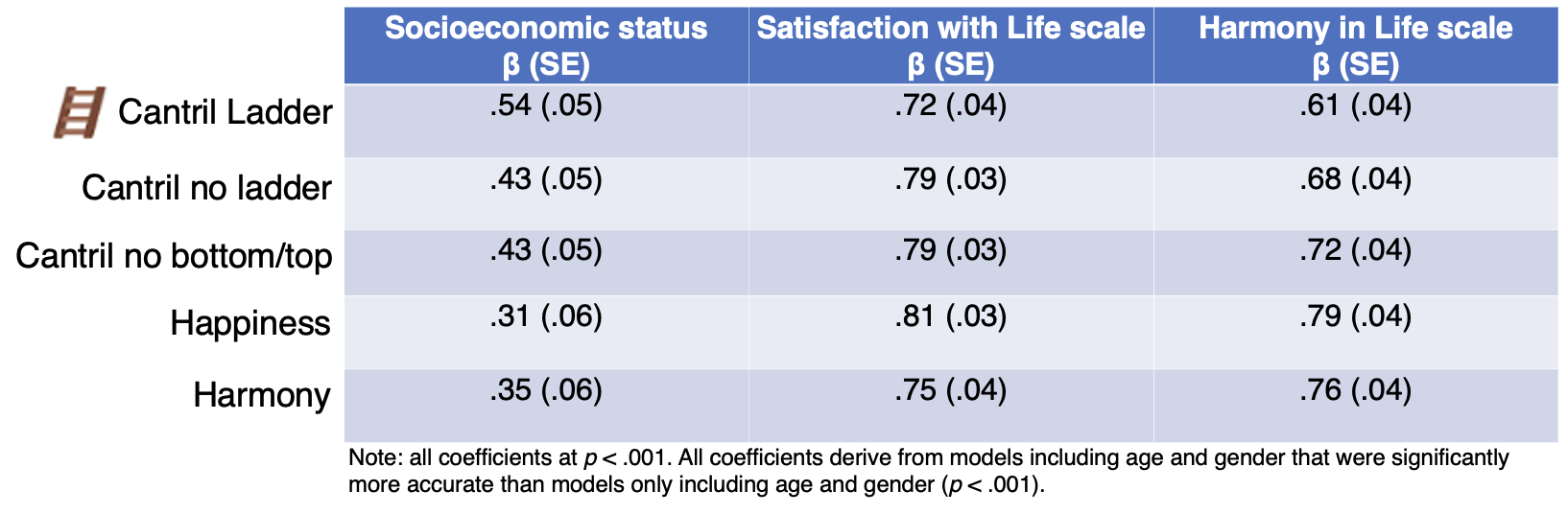
**

**Fig S8. Differences and frequencies of the word “family”**


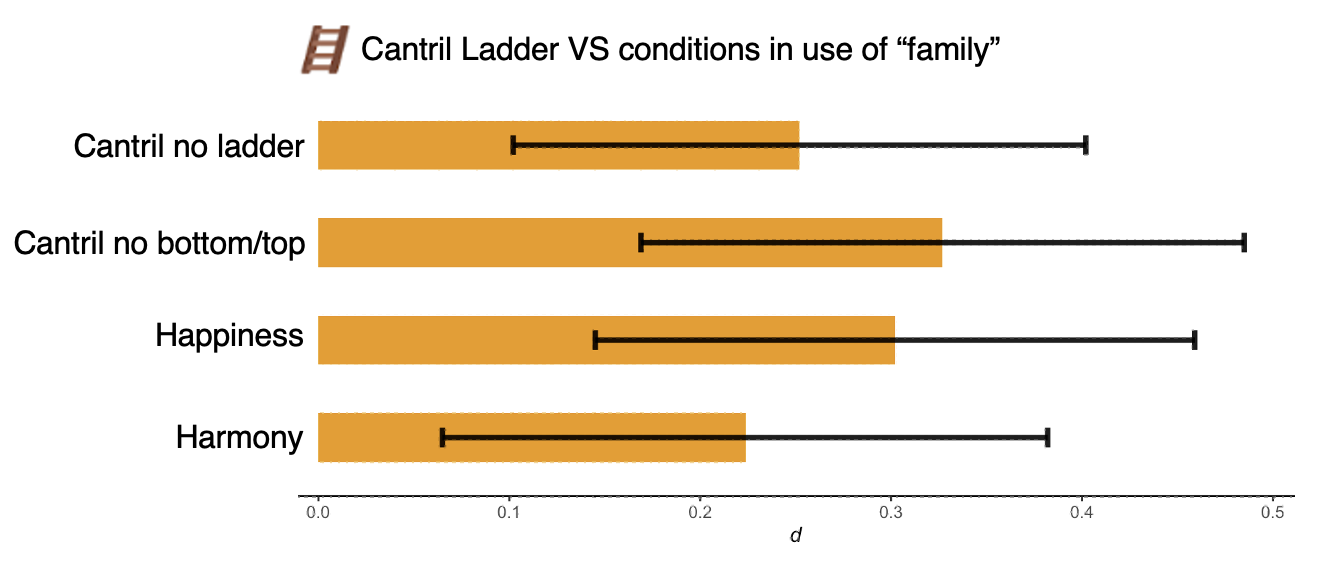


*Note:* all differences are significant compared to the Cantril Ladder (*p* < .05). Family was the most common word and analyzed separately to the topics.

**Fig S9. Summary and comparisons of the Language analyses approach**

**
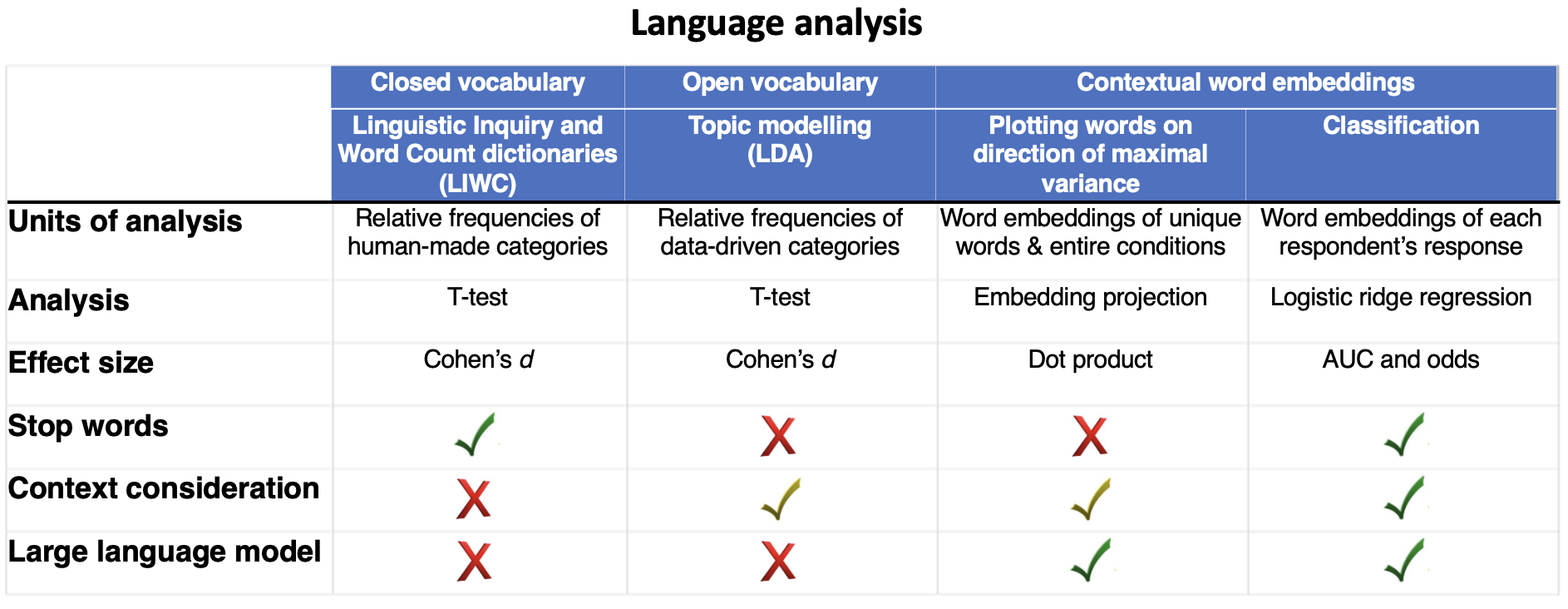
**

**Supplementary method**

**Attention check.** The survey included one attention check: “Please answer the alternative '4 neither agree nor disagree' below”. Participants who failed to answer it correctly were removed from further analysis. The included type of attention check has previously demonstrated increased reliability and statistical power in the data set (1).

**Harmony in Life and Satifaction with Life.** We used the two abbreviated three-item versions of the Harmony in Life Scale (HILS-3) and the Satisfaction with Life Scale (SWLS-3) to measure Harmony in Life and Satisfaction with Life, respectively (2), which derive from their five-item original (3,4). The scales include three items each (e.g., “I am in harmony” for the HILS-3, “I am satisfied with my life” for the SWLS-3) answered on Likert scales ranging from 1 to 7 (strongly disagree to strongly agree). Cronbach's Alpha and McDonald's Omega were both .94 for the HILS-3 and .91 for the SWLS-3.

For the statistical analyses, we used the LIWC software (5), the DLATK python package (6) that uses Mallet (7) and the following R-packages: *ggpubr* (8), *ggrepel* (9) *Hmisc* (10), *rstatix* (11), *psych* (12), *stringi* (13), *text* (14), *tibble* (15), *tidyverse* (16), *coin* (17), and *emmeans* (18).

**SI References**

1. Oppenheimer, D. M., Meyvis, T., & Davidenko, N. (2009). Instructional manipulation checks: Detecting satisficing to increase statistical power. *Journal of Experimental Social Psychology*, 45(4), pp. 867-872. DOI: 10.1016/j.jesp.2009.03.009
2. [Kjell, O. N. E., & Diener, E. (2021). Abbreviated Three-Item Versions of the Satisfaction with Life Scale and the Harmony in Life Scale Yield as Strong Psychometric Properties as the Original Scales. *Journal of Personality Assessment*, *103*(2), 183–194.](https://www.zotero.org/google-docs/?ZbX64j) <https://doi.org/10.1080/00223891.2020.1737093>
3. [Kjell, O. N. E., Daukantaitė, D., Hefferon, K., & Sikström, S. (2016). The Harmony in Life Scale Complements the Satisfaction with Life Scale: Expanding the Conceptualization of the Cognitive Component of Subjective Well-Being. *Social Indicators Research*, *126*(2), 893–919.](https://www.zotero.org/google-docs/?ZbX64j) <https://doi.org/10.1007/s11205-015-0903-z>
4. [Diener, E., Emmons, R. A., Larsen, R. J., & Griffin, S. (1985). The Satisfaction With Life Scale. *Journal of Personality Assessment*, *49*(1), 71–75. https://doi.org/10.1207/s15327752jpa4901_13](https://www.zotero.org/google-docs/?ZbX64j)
5. R. Boyd, A. Ashokkumar, S. Seraj, J. Pennebaker, “The development and psychometric properties of LIWC-22” (University of Texas at Austin, Austin, TX 2022). https://doi.org/10.13140/RG.2.2.23890.43205.
6. Schwartz HA, Giorgi S, Sap M, Crutchley P, Ungar L, Eichstaedt J, editor Dlatk: differential lan- guage analysis toolkit. *Proceedings of the 2017 Conference on Empirical Methods in Natural Language Processing: System Demonstrations*; 2017; Copenhagen, Denmark: Association for Computational Linguistics.
7. AK McCallum, Mallet: A Machine Learning for Language Toolkit. mallet.cs.umass.edu. (2002)
8. [Kassambara, A. (2020). *ggpubr: “ggplot2” Based Publication Ready Plots* (0.4.0) [Computer software].](https://www.zotero.org/google-docs/?ZbX64j) [https://CRAN.R-project.org/package=ggpubr](https://cran.r-project.org/package=ggpubr)
9. [Slowikowski, K., Schep, A., Hughes, S., Dang, T. K., Lukauskas, S., Irisson, J.-O., Kamvar, Z. N., Ryan, T., Christophe, D., Hiroaki, Y., Gramme, P., Abdol, A. M., Barrett, M., Cannoodt, R., Krassowski, M., Chirico, M., & Aphalo, P. (2021). *ggrepel: Automatically Position Non-Overlapping Text Labels with “ggplot2”* (0.9.1) [Computer software].](https://www.zotero.org/google-docs/?ZbX64j) [https://CRAN.R-project.org/package=ggrepel](https://cran.r-project.org/package=ggrepel)
10. Harrell, F. E. Jr., with contributions from Charles Dupont and many others. (2019). Hmisc:

Harrell Miscellaneous. *R package version 4.3-0*. [https://CRAN.R-project.org/package=Hmisc](https://cran.r-project.org/package=Hmisc)

1. [Kassambara, A. (2021). *rstatix: Pipe-Friendly Framework for Basic Statistical Tests* (0.7.0) [Computer software]. https://CRAN.R-project.org/package=rstatix](https://www.zotero.org/google-docs/?ZbX64j)
2. Revelle, W. (2021). psych: procedures for personality and psychological research.

*Northwestern University, Evanston, Illinois, USA*.

[https://CRAN.R-project.org/package=psych](https://cran.r-project.org/package=psych). Version = 2.1.9

1. Gagolewski, M. (2020). R package stringi: Character string processing facilities.

<https://cran.r-project.org/web/packages/stringi/index.html>

1. [Kjell, O., Giorgi, S., & Schwartz, H. A. (2021). *Text: An R-package for Analyzing and Visualizing Human Language Using Natural Language Processing and Deep Learning*. PsyArXiv.](https://www.zotero.org/google-docs/?ZbX64j) <https://doi.org/10.31234/osf.io/293kt>
2. Müller, K., Wickham, H., Francois, R., & Bryan, J. (2021). tibble: Simple Data Frames.

<https://cran.r-project.org/web/packages/tibble/index.html>

1. Wickham et al., (2019). Welcome to the tidyverse. *Journal of Open Source Software*, 4(43),

pp. 1686, DOI: 10.21105/joss.01686

<https://socialsciences.mcmaster.ca/jfox/Books/Companion/>

1. Hothorn T, Hornik K, van de Wiel MA, Zeileis A (2006). “A Lego system for conditional inference.” *The American Statistician, 60*(3), 257–263. Doi: 10.1198/000313006X118430.
2. Lenth, R., Singmann, H., Love, J., Buerkner, P. & Herve, M. Estimated marginal means, aka least-squares means. <https://cran.r-project.org/web/packages/emmeans/index.html>
